# Inflammatory response and oxidative stress during liver resection

**DOI:** 10.1371/journal.pone.0185685

**Published:** 2017-10-18

**Authors:** Christoph Schwarz, Fabian Fitschek, David Bar-Or, Daniel A. Klaus, Bianca Tudor, Edith Fleischmann, Georg Roth, Dietmar Tamandl, Thomas Wekerle, Michael Gnant, Martin Bodingbauer, Klaus Kaczirek

**Affiliations:** 1 Department of Surgery and Center for Perioperative Medicine, Medical University of Vienna, Vienna, Austria; 2 Section of Transplantation Immunology, Department of Surgery; Medical University of Vienna, Vienna, Austria; 3 Trauma Research Department, St. Anthony Hospital, Lakewood, Colorado, United States of America; 4 Trauma Research Department, Swedish Medical Center, Englewood, Colorado, United States of America; 5 Trauma Research Department, Medical Center of Plano, Plano, Texas, United States of America; 6 AYTU BioScience, Inc., Englewood, Colorado, United States of America; 7 Dept. of Anesthesiology, General Intensive Care and Pain Medicine; Medical University of Vienna, Vienna, Austria; 8 Department of Biomedical Imaging and Image Guided Therapy; Medical University of Vienna, Vienna, Austria; Medizinische Fakultat der RWTH Aachen, GERMANY

## Abstract

**Background:**

Postoperative complications are still a major concern after liver resection (LR). Systemic inflammation and deregulated reactive oxygen species during major abdominal surgery may impair outcome after hepatectomy.

**Methods:**

Patients undergoing LR were included in this study (n = 40). Oxidative stress (OS) was measured peri- and post-operatively as static oxidation-reduction potential markers (sORP) and antioxidant capacity ORP (cORP) by using the RedoxSYS Diagnostic system. Furthermore, Th1- and Th2-specific cytokines were assessed.

**Results:**

Whereas there was no significant change in systemic sORP during LR and in the early postoperative course, there was a substantial decrease of cORP immediately post-surgery, and on postoperative days 1 and 3 (p<0.001). OS response was tightly regulated, as there was a significant correlation between sORP and cORP (p<0.0001; R^2^:0.457). An increase of OS (sORP) after LR of more than 3 mV was predictive for severe postoperative complications (53.8% vs. 12.5; p = 0.017). There was a significantly higher IL-2 (p = 0.006) and IL-5 (p = 0.001) increase during hepatectomy in patients who developed a severe morbidity.

**Conclusion:**

Antioxidant capacity remained stable during LR but dropped during the post-surgical period, suggesting a consumption of antioxidants to maintain OS within healthy range. Severe postoperative complications were associated with a pronounced inflammatory response during surgery.

## Introduction

The liver is the second largest human organ orchestrating numerous functions in the human body including detoxification, hormone secretion, protein synthesis, and glycogen metabolism. Moreover, the liver plays a central role in the immune system. As a “first pass” organ, the liver filters blood from the splanchnic system, thus being a major contributor to the primary immune response to foreign antigens. [1–4] Liver resection (LR) is the only potential cure for a wide range of various diseases. As the liver has the potential to regenerate, up to 75% of the liver can be resected in healthy individuals with only a small risk for developing hepatic dysfunction. [5]. However, in spite of generally improved outcomes, LR is still associated with a substantial morbidity, accounting for a reduced oncological outcome probably as a consequence of a perpetuated inflammatory response, which contributes to immunosuppression. [6–9]

Oxidative stress (OS) is a result of an increased production of reactive oxygen species and a decrease in antioxidant enzymes such as superoxide dismutase, catalase, and reduced nicotinamide adenine dinucleotide peroxidase. This imbalance in oxidation-reduction potential (ORP) and the resulting damage play an important role in a wide range of physiological and pathological processes affecting all organs including the liver. [10, 11] Furthermore, oxidative stress plays a role in cancer progression and invasion [12] and metastasis through disrupting endothelial cell lining. [11] Conversely, it has also been suggested that OS might also limit metastasis and might therefore be oncologically beneficial. [13]

Immune homeostasis is tightly regulated and numerous factors contribute to a pronounced inflammatory state including obesity. [14–16] Major surgery is associated with a systemic response involving numerous pro- and anti-inflammatory cytokines. [17, 18] It has been proposed that increased inflammatory cytokine release is associated with adverse outcome after surgery. [18–20]

Therefore, we hypothesized that a pronounced systemic inflammatory response during LR might be involved in deterioration of postoperative outcome. The aim of this study was to investigate the systemic and local inflammatory response during LR, to explore the association between oxidative stress and surgical outcome following hepatectomy.

## Material and methods

This is a prospective observational study in patients undergoing LR at the Department of Surgery at the Medical University of Vienna. The study was reviewed and approved by the institutional review board of the Medical University of Vienna (IRB nb.: 1829/2012). Written informed consent was obtained from all participants.

### Patient population

Patients older than 18 years undergoing an elective hepatic resection including ≥ two segments of the liver were eligible for this study. Exclusion criteria were Hepatitis B, C or HIV infection, autoimmune disease, inflammatory bowel disease or pregnancy. Preoperative liver function was assessed by Child-Pugh score and indocyanine green clearance test (ICG) as described previously. [21]

Parenchymal transection was performed with stapler- or Cavitron Ultrasonic Surgical Asiprator (CUSA) LR. The types of LR were defined as major and minor resections according to the IHPBA Brisbane 2000 nomenclature (≤2 segments: minor; >2 segments: major). [22]

### Blood sampling

Blood samples were obtained pre-surgery (systemic), pre-resection (systemic, portal vein, hepatic vein), post-resection (systemic, portal vein, hepatic vein), post-surgery (systemic) and on post-operative days (POD) 1 and 3 (systemic). Blood samples were collected in pre-chilled Z Serum clot activator containing vacuum tubes. Samples were processed immediately after blood drawing. After centrifuging the samples at 1400 RPM (rounds per minute) for 10 min at 4°C, serum was stored at -80°C. Oxidative stress was measured by the RedoxSYS^®^ Diagnostic System in serum and was expressed as static oxidative reduction potential (sORP) and capacitiy ORP (cOPR) as previously described. [23] Cytokine levels were determined by using RayBio^®^ Quantibody Human Th1/Th2 Array 1 (RayBiotech, Norcross, GA, USA). Cytokine concentrations were assessed by RayBiotech Inc. by using their Quantibody service.

### Visceral fat

Visceral fat area at the level of L3, which has been shown to correlate well with total visceral fat volume was calculated in all patients who had a preoperative CT imaging (n = 30). Venous-phase axial images of the abdomen were exported to a workstation using OSIRIX V5.0 (Pixmeo, Sarl, Switzerland). A single slice on the level of L3, with both transverse processes visible, was selected. Semi-automated, specific tissue demarcation was performed using Hounsfield units (HU) between −150 and −50 for the delineation of visceral adipose tissue (VAT). Manual corrections were performed in case other structures outside the respective compartment were detected. The cross-sectional area of VAT was assessed.

### Study endpoints

Primary endpoint was the impact of oxidative stress during liver surgery on postoperative outcome. Secondary endpoints were perioperative inflammatory response including Th1 (IL-2, INF-ϒ) and Th2 (IL-4, IL-5) cytokines as well as other pro-inflammatory markers (IL-6, GM-CSF). Morbidity and mortality was assessed according to the Clavien-Dindo classification [24] within 30 days follow-up. Severe surgical complication was defined as a Clavien-Dindo score ≥ III. [25]

### Statistical analysis

Statistical analysis was performed using GraphPad Prism, version 6 (GraphPad Prism Software^®^, La Jolla, CA). Metric data were expressed as means with standard deviation (SD) or standard error of the mean (SEM) or as median with interquartile range (Q1-Q3) and analysis was performed with the Mann-Whitney U test or an unpaired t-test as indicated. The individual change in ORP was calculated using a paired t-test and correlations between metric variable were calculated by using the Pearson- or the Spearman test. Categorical values were compared with a Fishers-exact test or a chi-square test. A p-value of <0.05 was determined as the threshold for significance.

## Results

### Patient population

Baseline characteristics are shown in Table 1. Overall, 40 patients were analyzed for this study including 25 major and 15 minor LR. All patients were classified as Child Pugh score A (100%). The main reasons for hepatectomy were malignancies including colorectal cancer liver metastases (CLM), hepatocellular carcinoma (HCC) and metastases of other origin, followed by cystic echinococcosis. Ten patients (25%) developed a severe postoperative complication (Clavien Dindo ≥ III) after hepatectomy, including liver dysfunction (n = 2), portal vein thrombosis (n = 1), biliary leakage (n = 5) and wound dehiscence (n = 2) with a median time of detection of 7 days after surgery (range: 0–11). Notable, there was no significant difference regarding baseline characteristics between patients with or without severe postoperative complication (Table 1). Median length of ICU stay was 2.5 days (1–3.8) in patients with severe complication and 1 day (1–1.8) in patients without (p = 0.017). Furthermore, patients with a Clavien Dindo score > III had a significantly prolonged duration of their hospital stay (10 (8–11) days vs. 17 (14.3–31.8) days; p<0.0001).

**Table 1:** Patient characteristics and postoperative outcome.

Patient characteristics and outcome	Overall (n = 40)	No/minor complications (n = 30)	Severe complications (n = 10)	p-value
Age, median (Q1-Q3)	60.5 (44.7–71.3)	59.8	61.3	0.767
Sex [male], n (%)	21 (52.5)	16 (53.3)	5 (50)	1.000
Cause for LR*, n (%)				
CLM^†^	16 (40)	12 (40)	4 (40)	1.000
Echinococcosis	5 (12.5)	5 (16.7)	0	0.306
HCC^‡^	5 (12.5)	2 (6.7)	3 (30)	0.089
Adenoma	3 (7.5)	2 (6.7)	1 (10)	1.000
Others	11 (27.5)	9 (30)	2 (20)	0.696
Malignancy, n (%)	27 (67.5)	18 (60)	9 (90)	0.124
Preoperative CHT^§^, n (%)	18 (45)	13 (43.3)	5 (50)	0.731
Portal vein embolisation, n (%)	4 (10)	4 (13.3)	0	0.556
**ASA**^ǁ^ **grade, n (%)**				
Grade I	7 (17.5)	6 (20)	1 (10)	0.656
Grade II	13 (32.5)	10 (33.3)	3 (30)	1.000
Grade III	20 (50)	14 (46.7)	6 (60)	0.716
Grade IV	0	0	0	
**Child-Pugh Score, n (%)**				
A	40 (100)	30 (100)	10 (100)	1.000
B	0	0	0	
C	0	0	0	
**ICG**^¶^ **clearance pre surgery, median (Q1-Q3)**				
PDR^#^	25.2 (19.4–28.3)	24 (19.1–26.4)	28,5 (23.7–31.7)	0.093
R15**	2.3 (1.5–5.5)	2,4 (1.9–5.7)	1,4 (0.9–2.9)	0.117
**Type of liver resection, n (%)**				
Major LR	25 (62.5)	17 (56.7)	8 (70)	0.269
Minor LR	15 (37.5)	13 (43.3)	2 (20)	0.269
Surgery duration [min], median (Q1-Q3)	215.5 (163.8–271.3)	220 (161.3–270)	210 (174.8–260.3)	0.945
**Clavien-Dindo Grade, n (%)**				<0.0001****
I	3 (7.5)	3 (10)	0	
II	2 (5)	2 (6.7)	0	
IIIa	4 (10)	0	4 (40)	
IIIb	4 (10)	0	4 (40)	
IVa	1 (2.5)	0	1 (10)	
IVb	0	0	0	
V	1 (2.5)	0	1 (10)	
ICU ^††^stay, median (Q1-Q3)	1 (1–2)	1 (1–1.8)	2.5 (1–3.8)	0.017*
LOS^‡‡^, median (Q1-Q3)	11 (8.8–13.3)	10 (8–11)	17 (14.3–31.8)	<0.0001****

* liver resection,

^†^ colorectal liver metastasis,

^‡^ hepatocellular carcinoma,

^§^ chemotherapy,

^**ǁ**^ American Society of Anesthesiologists,

^**¶**^ Indocyanine green clearance,

^#^ plasma disappearance rate,

** retention rate at 15 min,

^††^intensive care unit,

^‡‡^ length of stay.

### Oxidative stress

**Fig 1:**
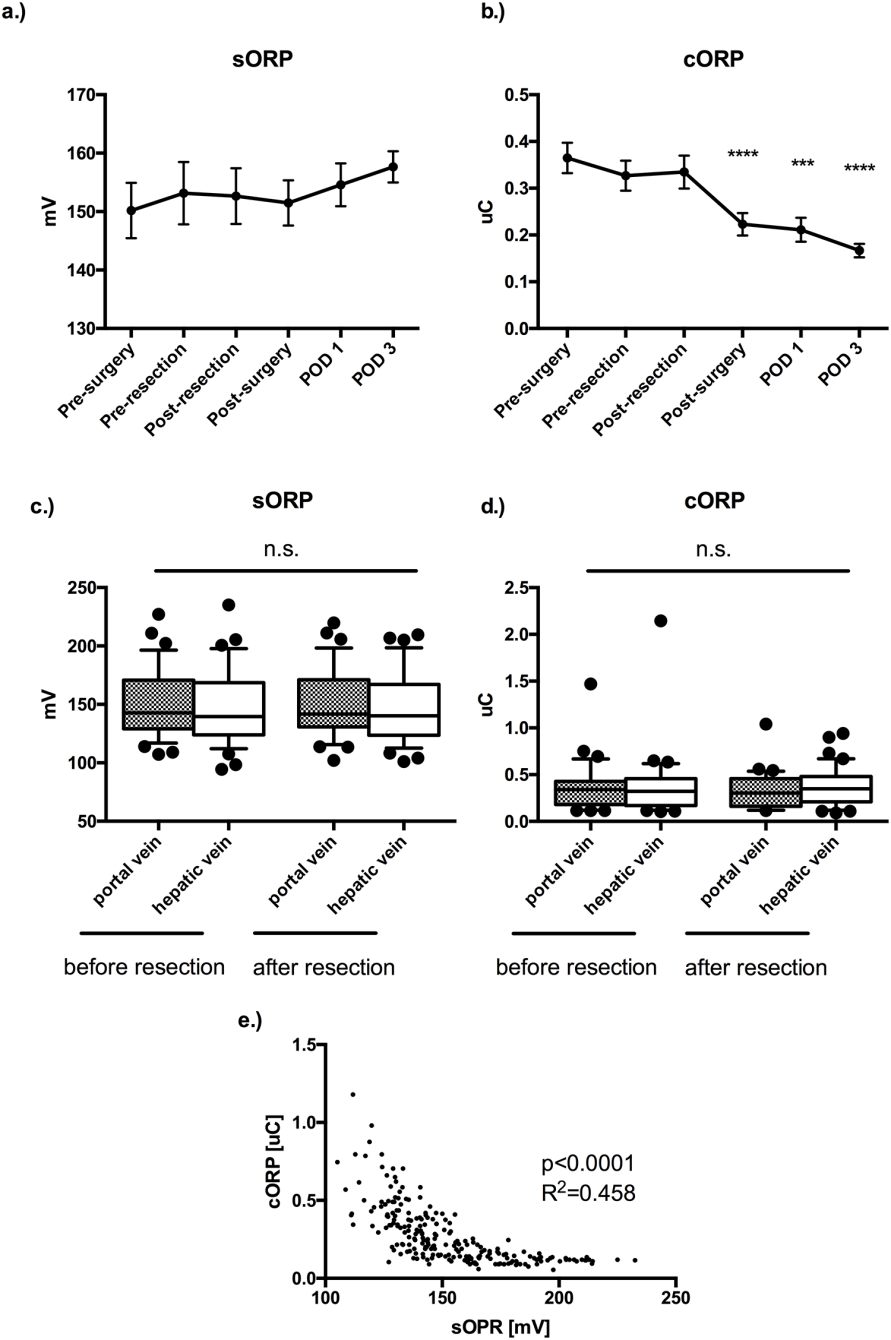
Oxidative stress (OS) during liver resection. **(A)** OS measured as static oxidative reduction potential (sORP) remains relatively stable during and after liver resection. **(B)** However, capacity oxidative reduction potential (cORP) significantly decreases after surgery and on postoperative day 1 and 3 (p<0.001). **(C, D)** Both, OS and capacity in the in- and outflow of the liver (portal vein, hepatic vein) remained virtually unchanged before and after liver resection, respectively. **(E)** OS is tightly regulated, as there was a significant correlation between sORP and cORP measured systemically (p<0.0001; R^2^: 0.458). Data are expressed as means with standard error of the mean (SEM) and comparison was performed by using a paired t-test (A, B) or an unpaired t-test (C, D). Correlation was calculated by using the Pearson-test (D). n.s. non significant, *** p<0.001, **** p<0.0001.

To assess the impact of LR on oxidative stress, systemic and locoregional (portal and hepatic vein before and after hepatectomy) serum samples were analyzed. Overall, there was no significant change in oxidative stress response measured as static oxidative reduction potential (sORP) during and immediately after LR (Fig 1a). However, whereas oxidative capacity (cORP) seemed to be stable perioperatively, there was a substantial decrease after surgery (1 b). Notably, both sORP and cORP from both the portal and the hepatic vein remained virtually unchanged during LR (Fig 1c and 1d). Patients with a very low remaining antioxidant capacity (cORP<0.11 uC) were significantly older than patients with higher values (median age: 72 vs. 59.2; p = 0.022). There was a significant correlation between sORP and cORP systemically at all measured time points (p<0.0001; R^2^: 0.457) (Fig 1e).

**Fig 2:**
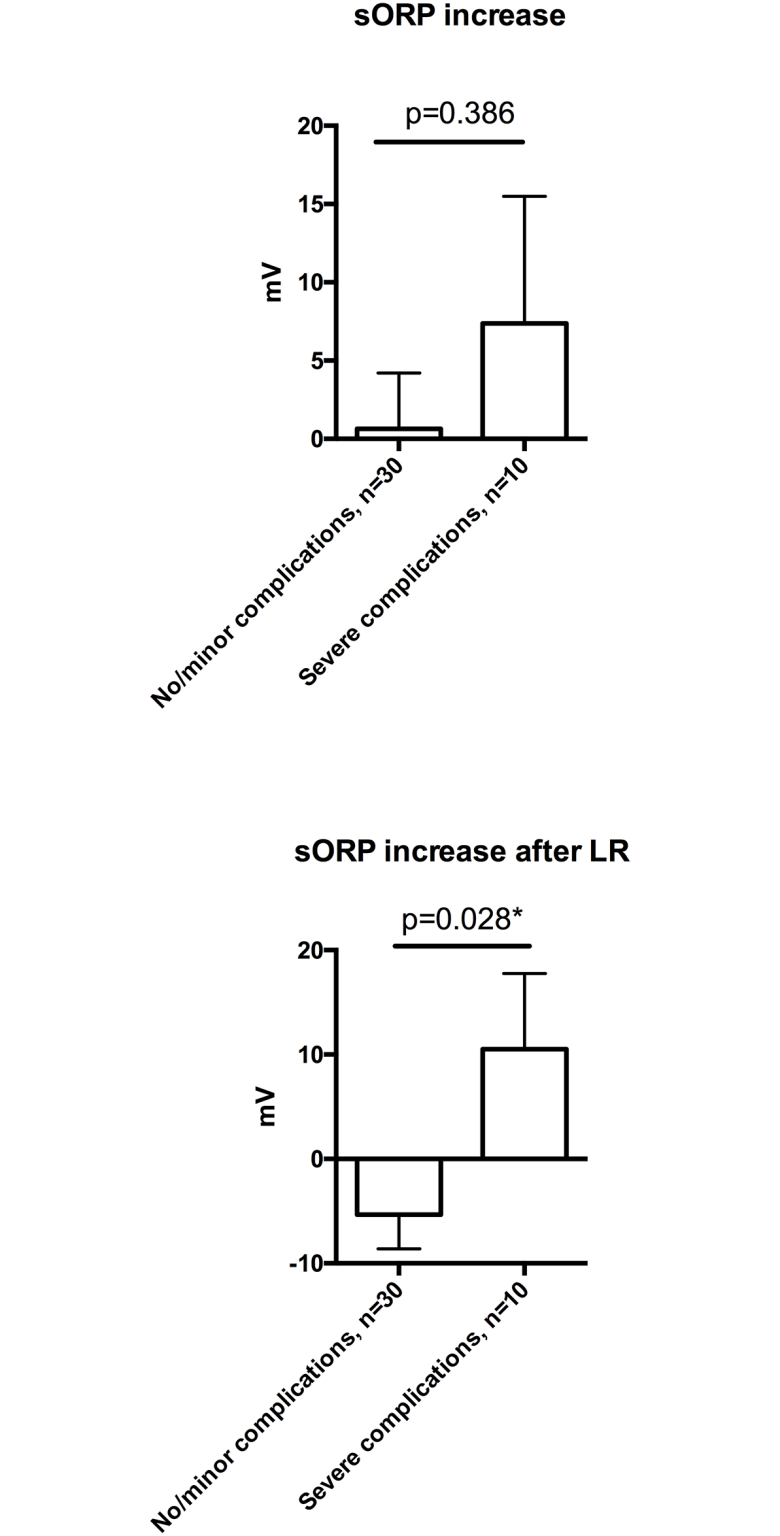
Change in OS is associated with severe post-operative complication. (A) Mean sORP increase during LR was higher in patients who developed a severe post-operative complication compared to patients with no/minor post-operative complication (0.6 (3.6) mV vs. 7.4 (8.1) mV; p = 0.386). (B) Measuring the increase of OS after LR revealed a significantly increased response in patients with severe morbidity compared to patients without (-5.3 (3.3) mV vs. 10.5 (7.3) mV; p = 0.028). Data are expressed as means with standard error of the mean (SEM) and comparison was performed by using an unpaired t-test.* p<0.05.

An increase of oxidative stress (sORP) after LR of more than 3 mV was predictive for severe postoperative complications (Clavien Dindo ≥ IIIa: 53.8% vs. 12.5 p = 0.017). Conversely, patients with severe postoperative complications had a higher increase of sORP (mean change: 10.5 mV vs. -5.3 mV; p = 0.028) (Fig 2).

### Inflammatory response

**Fig 3:**
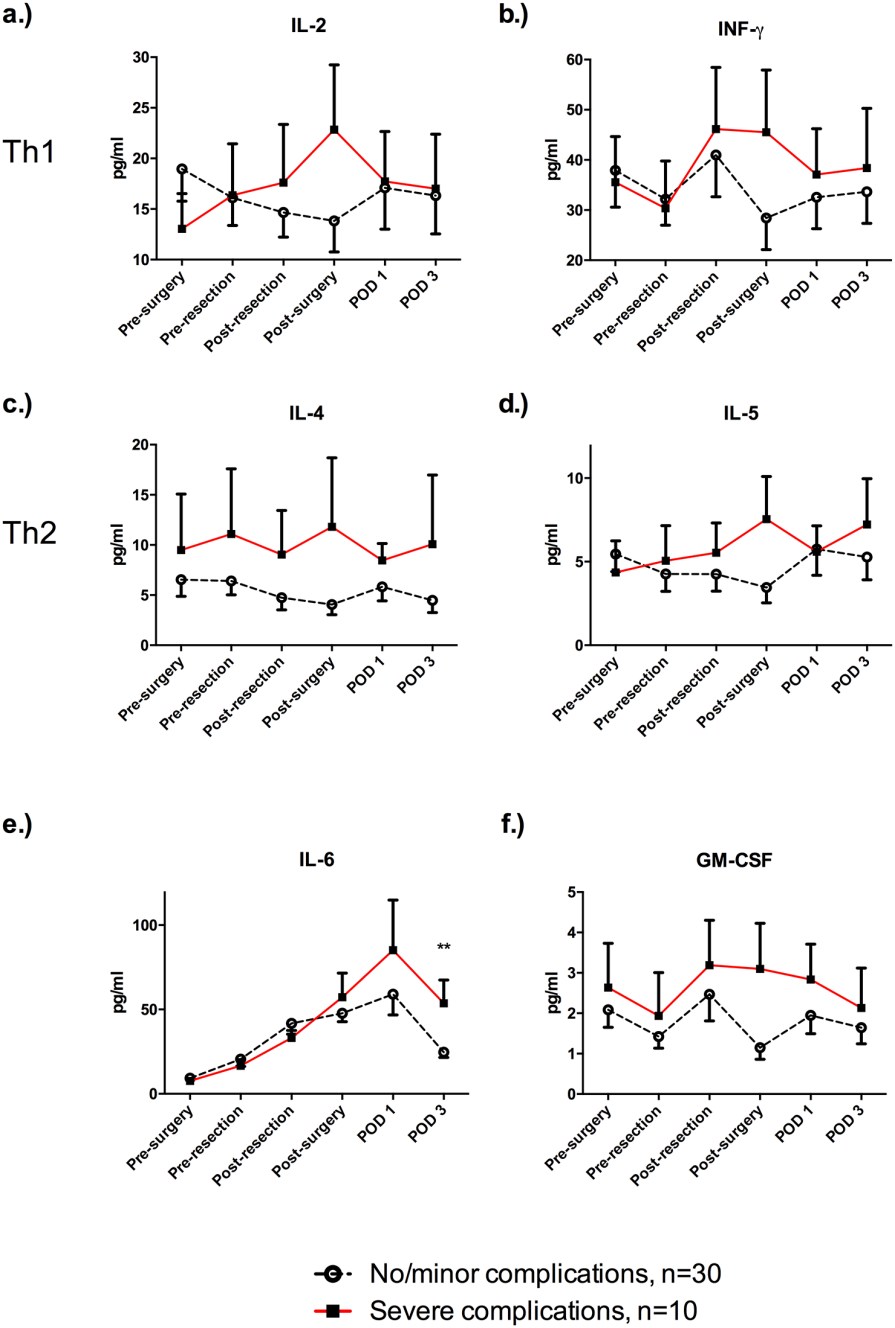
Cytokines during liver resection. Classical Th1 (A, B) and TH2 cytokines (C, D) were measured perioperatively. Even though there was a trend towards higher values of IL-2 (A), INF-y (B), IL-4 (C), IL-5 (D) and GM-CSF (F) perioperatively, the observed differences did not reach statistical significance. (E) Patients with severe post-operative complication had significantly higher levels of IL-6, measured POD3 (24.6 (3.1) pg/ml vs. (53.7 (13.8) pg/ml; p = 0.004). Data are expressed as means with standard error of the mean (SEM) and comparison was performed by using an unpaired t-test.** p <0.01.

Th1 (IL-2, INFy), Th2 (IL-4, IL-5) and inflammatory cytokines (IL-6, GMCSF) were measured in all patients during and after LR. Overall, there was a trend towards higher values in all measured cytokines in patients with a severe complication, although the observed difference did not reach statistical significance (Fig 3).

**Fig 4:**
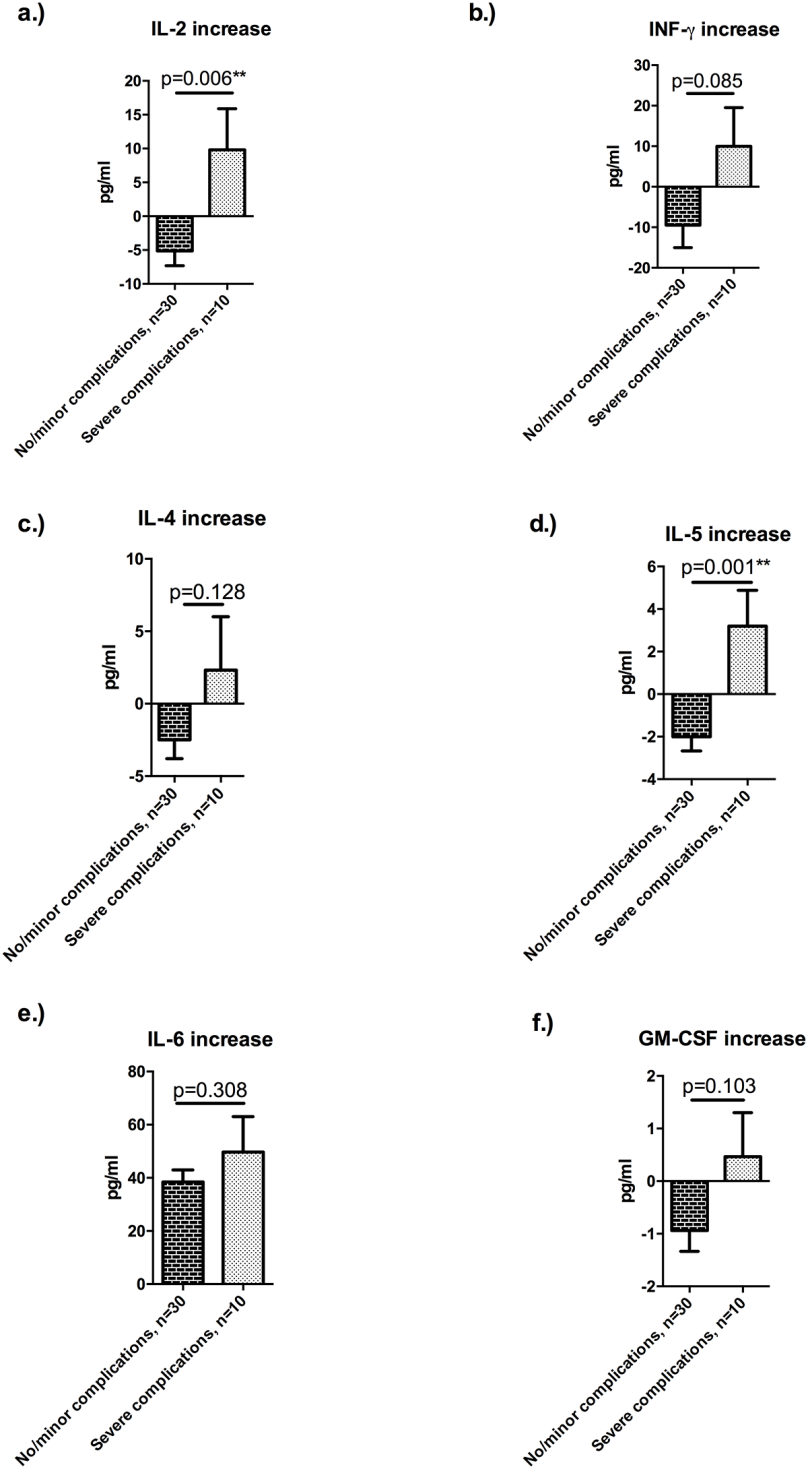
Inflammatory response during and after liver resection. **(A)** Mean interleukin-2 (IL-2) increase during surgery was more pronounced in patients, who developed severe postoperative complication (-5.1 (2.2) pg/ml vs. 9.8 (6.1) pg/ml; p = 0.006). **(B)** Similar to that, there was a trend towards an increased interferon-gamma (INF-y) response (p = 0.085). **(C, D)** Whereas there was only a trend towards a higher IL-4 increase during liver resection, an higher interleukin-5 (IL-5) increase during hepatectomy was predictive for severe postoperative complication (-2 (0.7) pg/ml vs. 3.2 (1.7) pg/ml; p = 0.001). **(E)** There was no significant difference regarding perioperative IL-6 increase. **(F)** Perioperative increase in granulocyte macrophage colony-stimulating factor (GM-CSF) was higher in patients with major complication compared to patients without, however the observed difference did not reach statistical significance. Data are expressed as means with standard error of the mean (SEM) and comparison was performed by using an unpaired t-test.** p<0.01.

Mean IL-2 increase during surgery was significantly higher in patients who developed a severe postoperative complication compared to patients who did not (-5.1 (2.2) pg/ml vs. 9.8 (6.1) pg/ml p = 0.006) (Fig 4a). Similar to that, there was a trend towards a higher INF-γ increase, however, the observed difference did not reach statistical significance (-9.5 (5.5) pg/ml vs. 10 (9.5) pg/ml; p = 0.085) (Fig 4b). Whereas there was only a tendency towards higher IL-4 levels in patients with postoperative complications (Fig 4c), there was a significantly higher perioperative IL-5 accumulation in the latter cohort (-2 (0.7) pg/ml vs. 3.2 (1.7) pg/ml; p = 0.001) (Fig 4d).

There was a considerable increase in IL-6 in all patients (Fig 4e). Notably, patients with severe complications had significantly higher levels of IL-6 on POD 3 compared to patients without complications (24.6 (3.1) pg/ml vs. (53.7 (13.8) pg/ml; p = 0.004). Overall, there was a trend towards higher GM-CSF levels in patients with severe postoperative complications. However, the observed difference did not reach statistical significance (Fig 4f).

As visceral obesity has been suggested to contribute to an increased pro-inflammatory response (14), we calculated VAT in all patients with preoperative CT scans (n = 30). Median visceral fat area was 113.15 cm^2^ (45.5–201.7) in all patients. There was a significant correlation between VAT and age (p = 0.003, r = 0.526). Further we found a significant connection between visceral obesity and perioperative increase in oxidative stress (p = 0.049, r = 0.363) (S1 Fig). Notably, there was no correlation between VAT and other inflammatory cytokines during or shortly after surgery.

## Discussion

Preventing severe postoperative complications remains an elusive goal in order to further improve outcome after LR. Herein we show that patients with severe morbidity have a significant increase in inflammatory markers and oxidative stress during surgery. This might be valuable for screening patients perioperatively with the intention to detect subjects who are at high risk for later developing severe postoperative complications.

Even though mortality has decreased substantially over the past decades, morbidity rates of liver surgery remain high between 20%-42%. [26–29] Surgical complications not only adversely impact on patient survival but also have a negative influence on oncological outcome probably due to perpetuated inflammation causing prolonged immunosuppression. [6–9] There are several well-described risk factors for patients to develop postoperative complications including age, major LR, the need for blood transfusion, high ASA scores or resection for malignancy. [30–32] In the present study, there was a trend towards higher age malignant underlying disease in patients with severe postoperative morbidity, however none of these differences reached statistical significance. As expected, outcome was significantly affected by the incidence of postoperative complications as both, length of ICU stay and overall length of hospital stay were significantly increased in patients with severe morbidity.

Our data show that antioxidant capacity remained stable during LR but dropped during the post-surgical period suggesting a consumption of antioxidant capacity to maintain oxidative stress within healthy range. Oxidative stress was tightly regulated, as there was a highly significant correlation between OS and oxidative capacity. We observed a significant association between postoperative outcome and perioperative inflammatory response including oxidative stress, Th1 (IL-2) and Th2 (IL-5) increase.

It has been shown previously that oxidative stress is closely associated with post-operative surgical complications. [33, 34] Ishikawa et al. observed a shift towards Th2 differentiation in patients with postoperative complications after major surgery on POD 14. Furthermore, patients with a complication had a significantly higher number of IL-4 producing CD4-T-cells on POD 2 [35] This is similar to our study, which revealed a trend towards higher IL-4 levels on the first POD even though the observed difference did not reach statistical significance. These results indicate that a pronounced immune response is connected with an increased risk for developing a severe post-operative morbidity.

There are two possible explanations for these observations: Firstly, patients who develop a severe complication might show an increased immune response as very early sign of postoperative morbidity. Secondly, a pronounced immune response might (at least partly) cause a pro-inflammatory state that leads to the occurrence of infections, wound healing disorders or impeded liver regeneration. This could be the explanation for the positive impact of preoperative steroids on liver function and complication in hepatic surgery. [36, 37]

In general, treatment options are limited for a number of morbidities including liver dysfunction. [38] We suggest, however, that patients with an intraoperative increase of inflammatory markers, who might be at high risk for developing a severe complication later, could benefit from prophylactic antibiotic therapy or closer monitoring at an ICU, interventions that would of course have to be validated prospectively.

Even though there was a significant correlation between VAT and age and a significant, but weak, correlation with change of perioperative oxidative stress, there was no connection to post-operative outcome, defined as morbidity or inflammatory response. We conclude that larger studies are needed to define the actual role of VAT in the inflammatory response during abdominal surgery.

There are several limitations that need to be considered when interpreting the results. The number of patients is relatively small for drawing general conclusions. However, the detailed analysis of locoregional and systemic inflammatory response might outweigh the latter limitations.

We conclude that the eventual “surgical” fate of a patient might already be determined during LR, and is reflected by a distinct inflammatory response. Further prospective studies are warranted in order to confirm the impact of inflammation and oxidative stress on postoperative outcome.

## Supporting information

S1 Fig Obesity and inflammatory response. **(A)** There was a significant correlation between visceral obesity measured in CT scans and perioperative increase in OS (p = 0.049, r = 0.363). (B) Furthermore, we found a significant connection between obesity and age (p = 0.003, r = 0.526). Correlation was calculated by using the Spearman-test.* p<0.05, ** p<0.01.
(TIFF)

S1 File Supporting data set. (XLSX)
