# Supplementary figures and images for: Inflammatory response and oxidative stress during liver resection
Source: PLoS One. 2017 Oct 18;12(10):e0185685. doi: 10.1371/journal.pone.0185685 (PMC5646773; doi:10.1371/journal.pone.0185685)

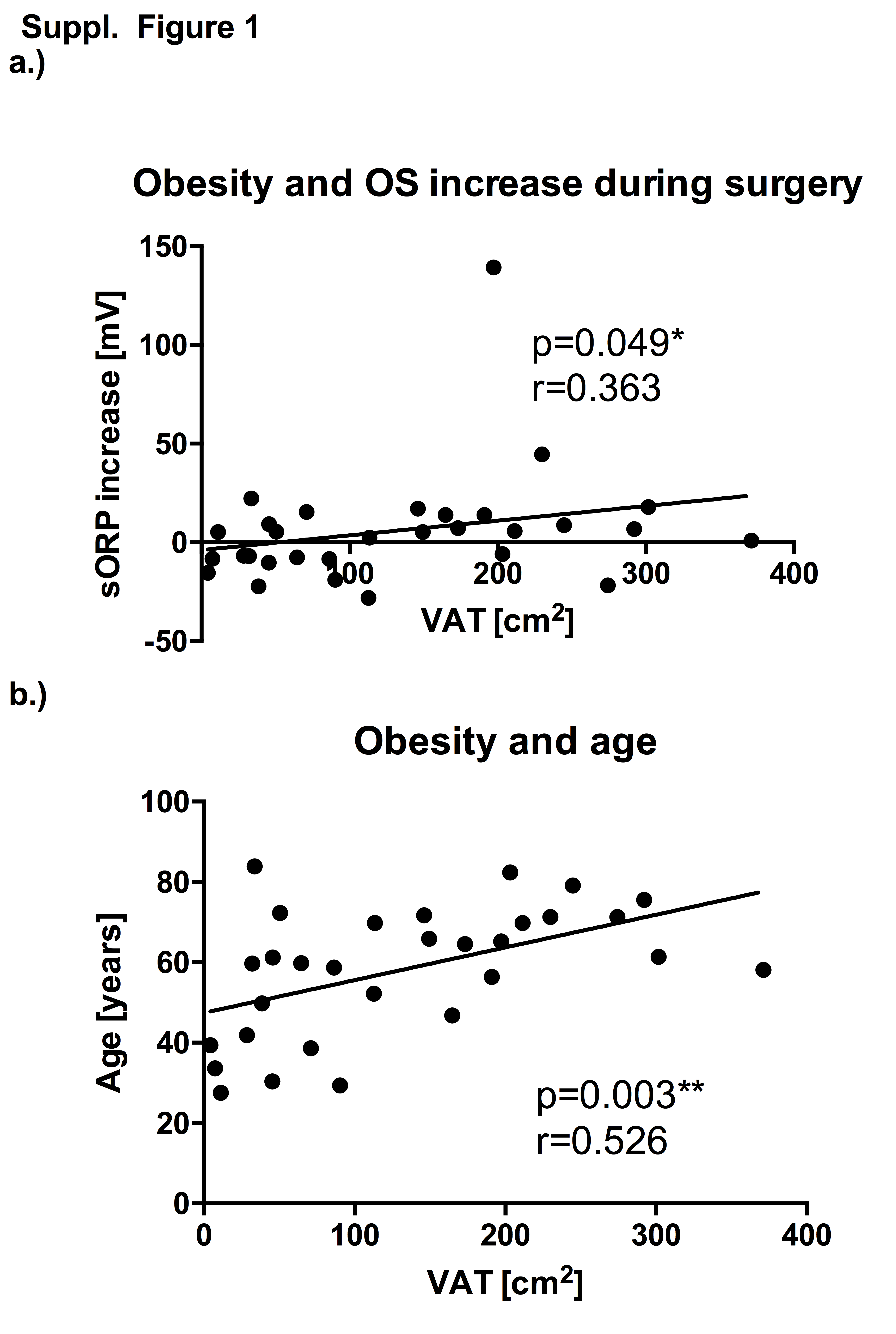

Supplement: S1 Fig — (A) There was a significant correlation between visceral obesity measured in CT scans and perioperative increase in OS (p = 0.049, r = 0.363). (B) Furthermore, we found a significant connection between obesity and age (p = 0.003, r = 0.526). Correlation was calculated by using the Spearman-test.* p<0.05, ** p<0.01. (TIFF) [file pone.0185685.s001.tiff]
